# Factors Associated with Survival and Discontinuation of Anti-Malarial Agents in Systemic Lupus Erythematosus: Results from a Tertiary Swedish Referral Centre

**DOI:** 10.3390/jcm13051485

**Published:** 2024-03-04

**Authors:** Tomas Walhelm, Lina Wirestam, Yvonne Enman, Ioannis Parodis, Christopher Sjöwall

**Affiliations:** 1 Division of Inflammation and Infection/Rheumatology, Department of Biomedical and Clinical Sciences, Linköping University, SE-581 83 Linköping, Sweden; lina.wirestam@liu.se; 2 Division of Rheumatology, Department of Medicine Solna, Karolinska Institutet, SE-171 77 Stockholm, Sweden; yvonne.enman@ki.se (Y.E.); ioannis.parodis@ki.se (I.P.); 3 Department of Gastroenterology, Dermatology and Rheumatology, Karolinska University Hospital, SE-171 64 Stockholm, Sweden; 4 Department of Rheumatology, Faculty of Medicine and Health, Örebro University, SE-701 82 Örebro, Sweden

**Keywords:** systemic lupus erythematosus, antimalarials, hydroxychloroquine, chloroquine, drug survival

## Abstract

**Background:** Antimalarial agents (AMAs) are cornerstone drugs in the treatment of systemic lupus erythematosus (SLE), and their use has established benefits, such as improved prognosis and decelerated accrual of organ damage. The aim of this study was to investigate the frequency of discontinuation of AMAs and associated factors in a Swedish SLE population. **Methods:** We retrieved data from a regional SLE register where all patients fulfilled the 1982 ACR and/or the 2012 SLICC classification criteria. A total of 328 subjects were included in the analysis. **Results:** Altogether, 92.4% (303/328) had been prescribed AMAs at some point during their disease. At the last available visit, 67.7% (222/328) were currently prescribed AMAs. Among individuals who had discontinued use, 24.7% (20/81) had developed a contraindication. Side effects were also common reasons for discontinuation (*n* = 38); gastrointestinal symptoms (52.6%, 20/38) were most common. Patients who discontinued had accrued more organ damage at the last visit (mean SDI: 2.9; SD: 2.8) compared with those still on AMAs (mean SDI: 1.4; SD: 1.8; *p* = 0.001). **Conclusions:** Most patients had been exposed to AMAs, but 25% discontinued therapy. Among side effects leading to discontinuation, >50% were gastrointestinal, calling for adequate gastroprotection towards drug retention and prevention of organ damage progression.

## 1. Introduction

Hydroxychloroquine (HCQ), chloroquine (CQ) and quinacrine, also referred to as antimalarial agents (AMAs), constitute backbone drugs in the treatment of systemic lupus erythematosus (SLE). HCQ is preferred, and most frequently used, due to its superior safety profile [1]. AMAs are weak bases and the mechanisms of action of AMAs in lupus are complex, including both innate and adaptive immune responses [2]. The main effects include inhibition of type I interferons released via suppression of endosomal Toll-like receptor activation and inhibition of the cyclic GMP-AMP synthase–Stimulator of Interferon Genes (cGAS-STING) pathway, but the full mechanisms of action are yet to be clarified [3]. AMAs can be used as monotherapy or in combination with other immunomodulatory drugs. The use of AMAs in SLE goes back more than 70 years and has been coupled with a multitude of beneficial effects, such as improved prognosis and decelerated accrual of organ damage [4].

AMAs are recommended for all patients with SLE according to the EULAR recommendations for the management of SLE, preferably at a daily dose that does not exceed 5 mg/kg and is also adjusted based on individual risk for adverse events [1]. However, use of AMAs is safe for most patients, and serious adverse effects are uncommon. During pregnancy, HCQ is safe and its use should be continued, but doses above 400 mg/daily are not recommended [5,6]. The most commonly reported side effects of AMAs are gastrointestinal symptoms, while the most serious side effect is retinal toxicity [7]. For this reason, regular retinal screening is recommended during use, especially after some years of accumulated exposure [8]. Management and prevention of flares is crucial in the treatment of chronic diseases. However, it is well-known that non-adherence to AMAs is an issue [9].

The aim of this study was to investigate the frequency of AMA prescription and identify factors that are associated with discontinuation of AMAs in an SLE patient population from Sweden, to guide management towards greater degrees of drug retainment. A similar study has been conducted in Spain, but never in Northern Europe [10].

## 2. Materials and Methods

### 2.1. Study Population and Data

We conducted this study based on data from the *Clinical Lupus Register in North-eastern Gothia* (Swedish acronym “KLURING”), a longitudinal research and quality register based at Linköping University Hospital, a tertiary Swedish referral centre, which offers highly specialised health-care services. KLURING includes practically all prevalent and incident cases of adult (≥18 years of age) SLE in Östergötland County from 2008 onwards [11]. We included 328 subjects with SLE in the statistical analysis. The patients were predominantly women (85.7%), and the mean age at the time of SLE diagnosis was 40.0 years (range: 3–85, standard deviation (SD): 17.7). All included subjects fulfilled the validated 1982 American College of Rheumatology (ACR) and/or the 2012 Systemic Lupus International Collaborating Clinics (SLICC) classification criteria and had been diagnosed with SLE from 1963 and onwards [12,13]. No exclusion criteria were applied.

Data regarding prescription of AMAs, pharmacological treatments, clinical phenotypes, adverse events and contraindications were obtained from medical records and KLURING protocol data. Organ damage was recorded annually using the SLICC/ACR damage index (SDI) [14].

### 2.2. Statistical Analysis

Factors associated with discontinuation of AMAs were investigated using logistic regression analysis, Mann––Whitney *U* test, Chi-squared (*χ*^2^) test, Fisher’s exact test and Wilcoxon signed-rank test for subgroup analyses. Factors that showed significant association in the univariable analysis were subsequently included in a multivariable model.

A subgroup analysis was performed to account for differences between the groups who discontinued AMAs and those who did not, whereby individuals from the register were matched for sex, age and disease duration. Factors contributing to adherence to the prescription were obtained from medical records and KLURING protocol data.

*p*-values <0.05 were considered statistically significant. Statistical analyses were performed using the SPSS software version 29.0.0.0 (SPSS Inc., Chicago, IL, USA).

## 3. Results

### 3.1. Study Population Characteristics

The mean number of fulfilled 1982 ACR criteria was 4.8 (range: 3–9) for the included patients [12]. As shown in Table 1, the most commonly fulfilled criterion was the presence of antinuclear antibodies (98.8%), followed by arthritis (77.4%). Patients in the study cohort had a mean disease duration of 16.9 years (1–58, SD: 11.3) at the time of the last visit, and the mean SDI score at the last visit was 2.0 (0–11, SD: 2.5).

In the KLURING register, 24.4% (80/328) of the patients had a history of treatment with biological drugs. Rituximab was the most used biological agent as 81.3% (65/80) of the patients had received at least one dose of rituximab. At the last available visit, 7.9% (26/328) of patients were on biologics, of whom 50% (13/26) were currently using belimumab. The most used antirheumatic therapy recorded at the last visit was mycophenolate mofetil (46/328, 14.9%). Detailed characteristics and treatments for all subjects included in the study are shown in Table 1.

### 3.2. Use of AMAs

**Figure 1:**
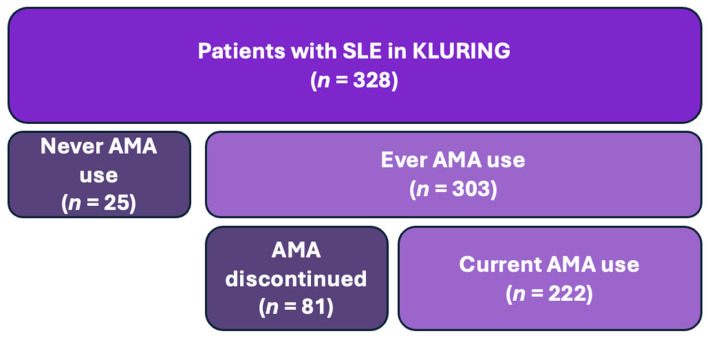
Schematic illustration of antimalarial agent (AMA) use and discontinuation in the KLURING cohort of patients with systemic lupus erythematosus (SLE).

In total, 92.4% (303/328) had been prescribed AMAs at some point during their disease course (Figure 1). Data from the last available visit for these patients indicated that 73.3% (222/303) were still prescribed AMAs. Factors preventing the initiation of AMA prescription included kidney disorders (*n* = 6/25), ophthalmologic disorders (*n* = 4/25), mild SLE (*n* = 3/25), patients’ preference (*n* = 3/25), liver disease (*n* = 2/25), psoriasis (*n* = 1/25) and unknown reasons (*n* = 6/25). AMA prescriptions were exclusively for HCQ and corresponded to a mean daily dose of 228.0 mg (range: 100–400, SD: 71.0). The most commonly used dose was 200 mg/day (79.7%), followed by 400 mg/day (13.1%). Of the patients with ongoing AMA treatment, 69.8% (155/222) used HCQ as a monotherapy (with or without glucocorticoids). We observed that patients meeting the immunological disorder ACR criterion (ACR-10) were more likely to continue using AMAs (*p* = 0.002). Table 2 summarises the proportions of AMA use and reasons for discontinuation.

### 3.3. AMA Discontinuation

In total, 26.7% (81/303) of the patients included in the study had discontinued their AMA treatment as of records at the last visit. As demonstrated in Figure 1 and Table 2, the most common drug-related factor leading to discontinuation was experience of side effects (46.9%, 38/81). The most frequently reported side effects were gastrointestinal symptoms (52.6%, 20/38), followed by skin (21.1%, 8/38) and neurological symptoms (18.4%, 7/38). Twenty of the eighty-one subjects who discontinued AMAs had developed a contraindication, mostly on an ophthalmological basis (30.0%, 6/20). The mean time from AMA initiation to the occurrence of an ophthalmological disorder was 16.3 years (range 10–19 years), and the recommendation to discontinue and the reason were stated by an ophthalmologist.

Other contraindications leading to discontinuation of AMAs included cardiac conditions (20.0%, 4/20; two patients due to heart failure and two patients due to cardiomyopathy diagnosed by a cardiologist) and renal failure (10.0%, 2/20).

Patients meeting the ACR criterion number 3 (photosensitivity) were more likely to develop an adverse event leading to discontinuation of AMAs (*p* = 0.05). The most common patient-related factor associated with discontinuation of AMAs was intentional non-adherence, e.g., low motivation (72.7%, 8/11). The reason for ending the prescription could not be ascertained for 12 individuals.

Patients who had discontinued AMAs had higher SDI scores at the last visit (mean: 2.9, SD: 2.8; mean disease duration at the last visit: 20.0 years; mean age: 66.9 years) compared with patients who still were on AMAs (mean: 1.4, SD: 1.8; mean disease duration at the last visit: 15.3 years; mean age: 55.3 years; *p* = 0.001). To account for the difference between the two groups regarding age and SLE duration, we also performed a subgroup analysis with patient groups who had (*n* = 81) or had not (*n* = 81) discontinued AMAs; yielding similar age (mean age: 66.9 years; SD: 15.2 vs. mean age: 66.2 years; SD: 15.3, respectively), sex (female/male patients: 70/11 and 70/11, respectively) and SLE duration (mean: 20.0 years; SD: 11.8 and mean: 19.6 years; SD: 11.0, respectively). In this analysis, the group who discontinued AMAs still had higher SDI scores at the last visit (mean: 2.9, SD: 2.8) compared with the subgroup of patients who remained on AMA treatment (mean: 1.6, SD: 1.6; *p* = 0.001).

We observed that patients meeting the ACR criterion number 2 (discoid lupus) were more likely to discontinue AMA use (*p* = 0.04). Patients with a higher age at the time of SLE diagnosis, and as of records at the last visit, were found to be more likely to discontinue use of AMAs (*p* = 0.002 and *p* = 0.001, respectively). Patients with a longer follow-up time also demonstrated an increased likelihood to discontinue AMA therapy by the end of the study follow-up (*p* = 0.001).

Logistic regression analysis was performed to ascertain the effects of age at diagnosis, disease duration and SDI score at the last visit, the ACR criterion number 2 (discoid lupus) and the ACR criterion number 10 (immunological disorder) on the likelihood to discontinue AMA therapy. The logistic regression model was statistically significant, *χ^2^* (5) = 38.900, *p* = 0.001. Table 3 details the results from the univariable and multivariable logistic regression analysis of factors investigated for their association with AMA discontinuation.

## 4. Discussion

This study demonstrates that, while most patients with SLE in this geographical region of Sweden are provided with prescriptions of AMAs during follow-up, a substantial proportion of patients discontinue therapy. The main reason for discontinuation was drug-related factors, such as the development of contraindications or experienced side effects. More than half of the reported side effects leading to AMA discontinuation were gastrointestinal symptoms. Notably, patients who had discontinued AMA therapy as of records at the last follow-up visit appeared to have accrued more organ damage over time until the end of study follow-up, compared with patients who still were on AMAs. This is in line with previous observations from several groups [7,15]. Importantly, this difference remained significant even after adjustments for sex, age and SLE duration.

Use of AMAs is recommended for all patients with SLE, except for patients with apparent contraindications [1]. In our study population, the percentage of patients who had continued with their AMA therapy as of records at the last follow-up visit was relatively high (approximately 75%). These results are in conformity with two Japanese studies and a Spanish study with a long-term follow-up, which investigated continuation frequencies of HCQ therapy in SLE [10,16,17]. These studies reported similar patterns of adverse events, with gastrointestinal symptoms and skin rash being the most common ones, yet with a somewhat higher frequency of experienced adverse events. Importantly, re-administration or lowering of the dose of AMAs was often successful in helping the patients continue the antimalarial treatment [16]. As in the current study, a limitation in the Japanese studies was that blood concentrations of HCQ were not measured. To enhance the coverage of AMA use among SLE populations, it appears important that the prescriber is attentive and continuously informs patients about the possibility of side effects and adjusts the prescribed dose to the patient’s individual needs and comorbidity profile, in order to enhance the probability of continuing use of the drug [18].

One of the major concerns with AMA use is retinopathy and a low, yet existing and distressing, risk for permanent visual loss [7]. Treatment duration and dosage are main factors contributing to retinal toxicity; the risk for retinopathy is low during the first five years of HCQ use [8]. Ocular safety is a high priority; hence, regular ophthalmological controls are recommended at baseline and yearly from the fifth year of therapy onwards [1]. Importantly, in one third of the patients who discontinued their AMA therapy due to contraindications in the present study, the reason for discontinuation was occurrence of ocular adverse conditions. Time from initiation of AMA treatment to development of retinal conditions was nearly two decades of use in mean.

Long-term treatment with AMAs is associated with decreased disease activity, reduced risk for flares, including renal flares, and enhanced survival [19,20]. Our study demonstrates that patients in the KLURING register who remained on AMA therapy as of records at the last follow-up visit had accrued less organ damage compared with patients who had discontinued their AMA therapy. This association between AMA discontinuation and organ damage accrual remained significant in a subgroup analysis with matched individuals in the two groups (patients who discontinued AMA therapy and those who did not) in terms of age, sex and disease duration until the end of follow-up. By contrast, a multicentre retrospective study could not confirm an increased risk for flares in SLE patients over 55 years of age with stable disease who discontinued AMA use compared with matched patients who did not; however, the number of patients in that study was low, limiting the reliability of statistical calculations [21].

Our data showed a trend indicating that patients of a higher biological age were more likely to discontinue AMA therapy. This is in line with the observation by Araújo et al. [10]. One explanation for this observation could be the presence of a greater number of accumulated and more complex comorbidities in older patients compared with younger patients with SLE. Furthermore, a similar pattern was also observed for age at the time of SLE diagnosis; patients with a higher age were more likely to discontinue AMAs by the end of the study follow-up. Acknowledging the fact of substantial diagnostic delays for SLE patients [22] and considering the multitude of beneficial and protective effects of AMA therapy in SLE, early initiation of AMA treatment as soon as the SLE diagnosis is made appears important to increase the likelihood of an enhanced prognosis and in the prevention of an accrual of organ damage in this patient population.

Our study has some limitations. Renewal of prescriptions of AMAs is not equal to drug uptake; due to the retrospective nature of the study, we were unable to evaluate the degree of medication adherence, e.g., with determination of blood concentrations of HCQ. The cost of healthcare and medicinal treatment may contribute to non-adherence [18]; however, healthcare in Sweden is tax-funded, and universal access to prescribed drugs is provided with a high-cost protection system, i.e., patients are provided access to healthcare and to medications free of charge above a certain yearly amount of money spent for those purposes. In our study population, almost nine out of ten patients were women, which is in accordance with general estimations of sex distributions in SLE [23]. Furthermore, there was no significant differences in results between the female and the male SLE subgroups of the SLE population in our study. A major strength of our study was that the KLURING register comprises practically all cases of SLE in Östergötland County in Sweden, which increases the reliability of the results for the patient population of this region [11]. To our knowledge, this is the first study to address factors contributing to discontinuation of AMA therapy and the association between discontinuation of AMA therapy and organ damage progression in a Scandinavian population of SLE patients.

## 5. Conclusions

In the SLE population from a tertiary referral centre in mid Sweden investigated herein, the vast majority of patients had been prescribed AMAs, but about one quarter discontinued their AMA therapy during follow-up. The main reasons for discontinuation included development of contraindications or side effects. More than 50% of the reported side effects leading to discontinuation of AMA therapy were gastrointestinal symptoms, suggesting that gastroprotection upon report of such symptoms may help decrease AMA discontinuation rates. Our data are also suggestive of a protective effect of AMAs against organ damage accrual.

## Figures and Tables

**Table 1:** Characteristics of the included patients.

Patient Characteristics	*n*, Mean (Range or %)
Background variables	
Women	281 (85.7)
Age at diagnosis, mean (range), years	40.0 (3–85)
SDI score at last visit, mean (range)	2.0 (0–11)
Biologics, use ever	80 (24.4)
Biologics, at last visit	26 (7.9)
Number of fulfilled 1982 ACR criteria, mean (range)	4.8 (3–9)
Clinical phenotypes *	*n* (%)
Malar rash	123 (37.5)
Discoid lupus	50 (15.2)
Photosensitivity	170 (51.8)
Oral ulcers	39 (11.9)
Arthritis	254 (77.4)
Serositis	116 (35.4)
Renal disorder	88 (26.8)
Neurological disorder	20 (6.1)
Haematological disorder	202 (61.6)
Immunological disorder	181 (55.2)
Antinuclear antibody (immunofluorescence microscopy)	324 (98.8)
Antirheumatic therapy ^#^	*n* (%)
Mycophenolate mofetil	46 (14.9)
Methotrexate	28 (8.5)
Azathioprine	15 (4.6)
Belimumab	13 (4.0)
Rituximab	10 (3.0)
Sirolimus	7 (2.1)
Cyclosporine	3 (0.9)
Abatacept	1 (0.3)
Anifrolumab	1 (0.3)
Baricitinib	1 (0.3)
Leflunomide	1 (0.3)
Tacrolimus	1 (0.3)

* 1982 ACR criteria definitions [12]; ^#^ At the last visit.

**Table 2:** Antimalarial therapy and reasons for discontinuation.

Antimalarial Therapy					*n* (%)		
	Antimalarials, users (ever)				303 (92.4)		
	Antimalarials, current users				222 (73.3)		
		Hydroxychloroquine, %, daily dose in mg, mean (range)			100%, 228.0 (100–400)		
	Antimalarials, users who discontinued				81 (26.7)		
		Drug-related factors			58 (71.6)		
			Related to contraindication			20 (24.7)	
				Ophthalmological conditions (%)			(30.0)
				Cardiac conditions (%)			(20.0)
				Renal failure (%)			(10.0)
				Psoriasis (%)			(10.0)
				Liver conditions (%)			(5.0)
				Other, unspecified (%)			(25.0)
			Related to adverse events ^1^			38 (46.9)	
				Gastrointestinal symptoms (%)			(52.6)
				Dermatological symptoms (%)			(21.1)
				Neurological symptoms (%)			(18.4)
				Ophthalmological symptoms (%)			(13.2)
				Musculoskeletal symptoms (%)			(5.3)
				Psychiatric symptoms (%)			(5.3)
		Patient-related factors			11 (13.6)		
			Low motivation (%)			(72.7)	
			Low treatment expectations (%)			(18.2)	
			Anxiety about possible adverse events (%)			(9.1)	
		Unknown reason			12 (14.8)		

^1^ Some users experienced more than one adverse event.

**Table 3:** Univariable and multivariable analysis of factors investigated for their association with survival and discontinuation of antimalarial agents.

Univariate Analysis of Factors			Continuing AMA (*n* = 222)	Discontinuing AMA (*n* = 81)	*p*-Value
Gender					
Women, *n*, (%)			192 (86.5)	70 (86.4)	NA
Men, *n*, (%)			30 (13.5)	11 (13.6)	NA
Age at diagnosis, mean, (SD), years			38.1 (17.7)	44.8 (16.0)	**0.002**
Age at last visit, mean, (SD), years			55.3 (10.5)	66.9 (15.2)	**0.001**
Disease duration, years, mean (SD), years			15.3 (10.5)	20.0 (11.8)	**0.001**
SDI score at last visit, mean (SD)			1.4 (1.8)	2.9 (2.8)	**0.001**
Biologics, use ever, *n*, (%)			55 (24.8)	18 (22.2)	0.6
Biologics, at last visit, *n*, (%)			22 (9.9)	4 (4.9)	0.2
Ever smoker, *n*, (%)			92 (41.4)	38 (46.9)	0.2
Clinical phenotypes, *n*, (%) *					
Malar rash			84 (37.8)	26 (32.1)	0.4
Discoid lupus			28 (12.6)	18 (22.2)	**0.04**
Photosensitivity			118 (53.2)	41 (50.6)	0.7
Oral ulcers			26 (11.7)	10 (12.3)	0.9
Arthritis			176 (79.3)	66 (81.5)	0.7
Serositis			80 (36.0)	27 (33.3)	0.7
Renal disorder			58 (26.1)	14 (17.3)	0.1
Neurological disorder			10 (4.5)	6 (7.4)	0.4
Haematological disorder			136 (61.3)	49 (60.5)	0.9
Immunological disorder			132 (59.5)	32 (39.5)	**0.002**
Antinuclear antibody			219 (98.6)	80 (98.8)	0.9
**Multivariate analysis of factors** (Logistic regression analysis)					
**Factor**	**B**	**S.E.**	**Odds Ratio**	**95% C.I.**	***p*** **-Value**
Age at diagnosis	−0.020	0.010	0.981	0.962–0.999	**0.041**
Disease duration (years)	−0.030	0.015	0.971	0.942–1.000	0.051
SDI score at last visit	−0.179	0.070	0.836	0.729–0.959	**0.011**
Discoid lupus	0.394	0.366	1.483	0.724–3.039	0.282
Immunological disorder	−0.540	0.285	0.583	0.333–1.019	0.058

NA—not applicable; * 1982 ACR criteria definitions [12].

## Data Availability

The data given in this article are the datasets analysed during the study and are available from the corresponding author on reasonable request.
